# Surgical management for achalasia after coronary artery bypass graft using the right gastroepiploic artery: a case report

**DOI:** 10.1186/s40792-017-0300-8

**Published:** 2017-02-14

**Authors:** Ryo Muranushi, Tatsuya Miyazaki, Hideyuki Saito, Kengo Kuriyama, Tomonori Yoshida, Yuji Kumakura, Hiroaki Honjyo, Takehiko Yokobori, Makoto Sakai, Makoto Sohda, Hiroyuki Kuwano

**Affiliations:** Department of General Surgical Science, Gunma University Graduate School of Medicine, Gunma University, 3-39-22, Showa-machi, Maebashi, Gunma 371-8511 Japan

**Keywords:** Achalasia, Coronary artery bypass graft, Right gastroepiploic artery

## Abstract

**Background:**

The right gastroepiploic artery is commonly used in coronary artery bypass grafting. Appropriate strategies are required when performing upper abdominal surgeries after the right gastroepiploic artery has been used in coronary artery bypass grafting because compressing or injuring the graft may cause myocardial ischemia and fatal arrhythmias. To our knowledge, this is the first reported case of surgery for achalasia performed after coronary artery bypass grafting using the right gastroepiploic artery. We have discussed the surgical procedure and particular intraoperative considerations.

**Case presentation:**

A 62-year-old man who had undergone coronary artery bypass grafting using the right gastroepiploic artery presented with achalasia. Because medication and balloon dilation had been ineffective and he was having difficulty ingesting food, we performed a Heller–Dor procedure via laparotomy. The right gastroepiploic artery was not damaged during this surgery, and there were no perioperative cardiovascular complications. Adequate control of symptoms was achieved.

**Conclusions:**

When performing upper abdominal surgeries after coronary artery bypass grafting with the right gastroepiploic artery, it is necessary to investigate the patient carefully preoperatively and adapt the intraoperative procedure to minimize risk of injury to the graft and consequent cardiovascular complications.

## Background

The right gastroepiploic artery (RGEA) is sometimes used in coronary artery bypass grafting (CABG) as an alternative source of an arterial graft. It is recognized as a reliable conduit with superior long-term patency and is easy to anastomose to the right coronary artery [1]. According to a report by the Japanese Association for Coronary Artery Surgery, CABG was carried out in more than 0.1 million patients over the 7 years ending in 2004 and the RGEA was used in more than half of the surgeries involving anastomosis to the right coronary artery [2]. With improvements in prognosis after CABG, the frequency of abdominal surgery in patients who have undergone CABG using the RGEA is increasing. In such procedures, appropriate strategies are required because compressing or injuring the graft may cause myocardial ischemia and fatal arrhythmias.

Achalasia is a rare disease, its incidence being approximately 1/100,000 annually and its prevalence 10/100,000 [3]. Although there are some reports about gastrectomy for gastric cancer after CABG using RGEA graft and about simultaneous esophagectomy and CABG for esophageal cancer [4], to our knowledge, there are no published reports about surgery for achalasia in this context. We here present a case in which we performed surgery for achalasia after CABG using the RGEA.

## Case presentation

**Fig. 1:**
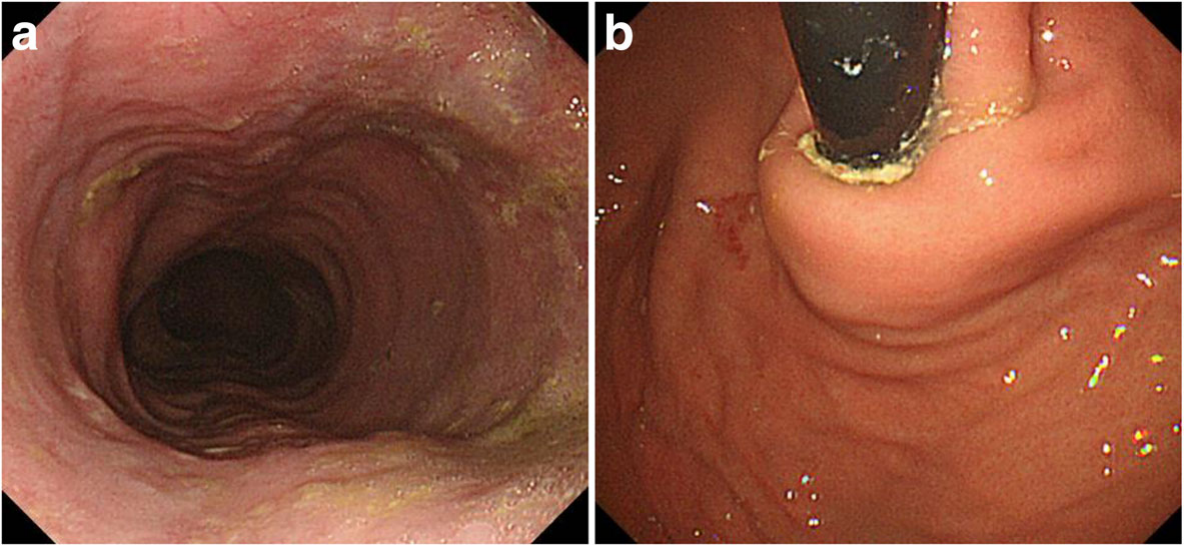
Gastrointestinal endoscopy findings. **a** The esophagus is dilated and meandering and contains food residues. **b** The scope has twisted and is eccentric in the cardiac region, a characteristic finding of achalasia

**Fig. 2:**
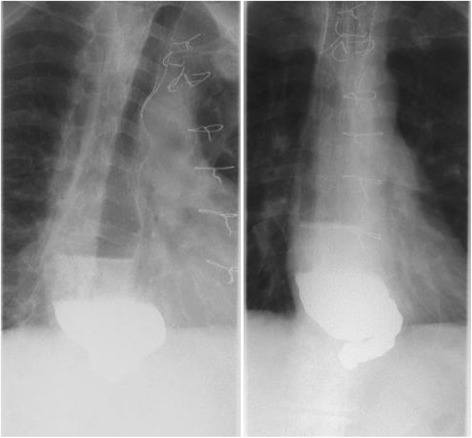
Barium esophagogram findings. The esophagus is dilated and meandering and barium outflow to the stomach is delayed

**Fig. 3:**
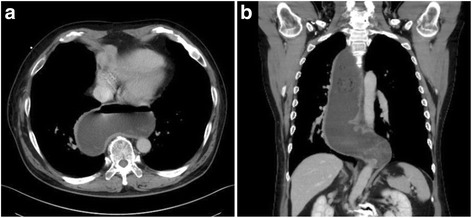
Enhanced CT findings. The esophagus is dilated and the esophagus is meandering, particularly in its inferior portion. **a** Axial view. **b** Coronal view

**Fig. 4:**
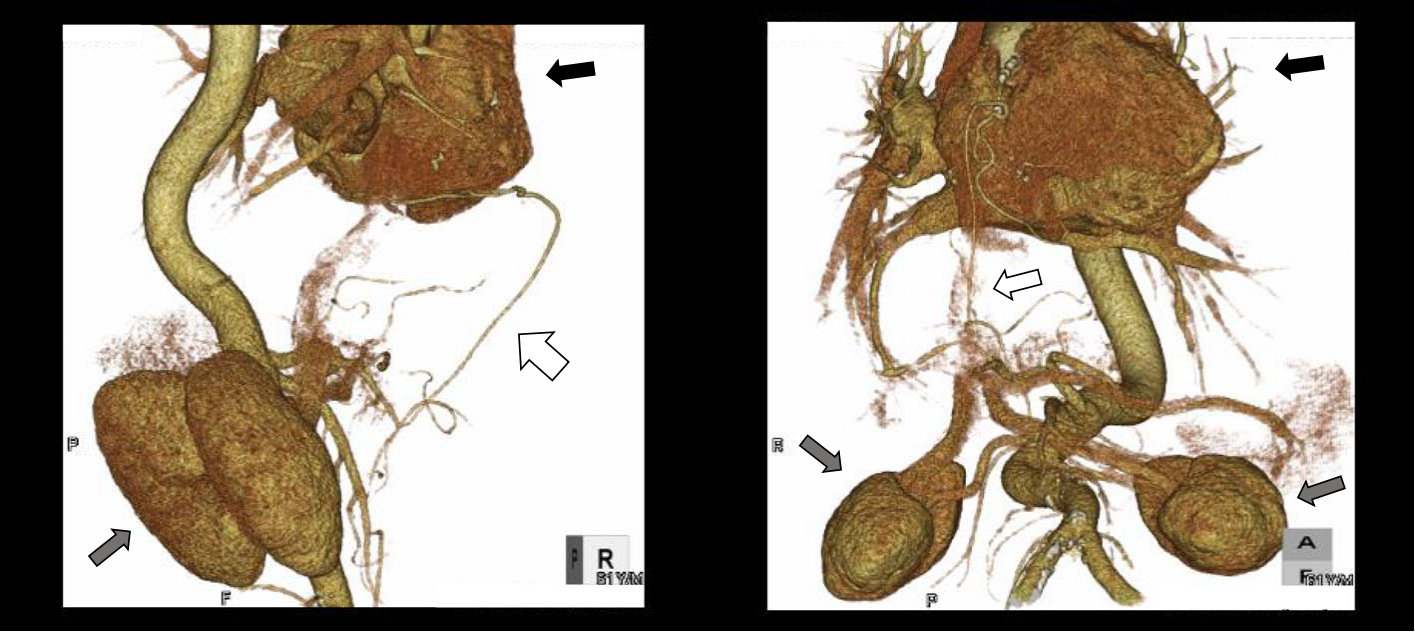
Three-dimensional angiography findings. The right gastroepiploic artery is running into the right coronary artery. *White arrow* right gastroepiploic artery, *black arrow* heart, *gray arrow* kidney

**Fig. 5:**
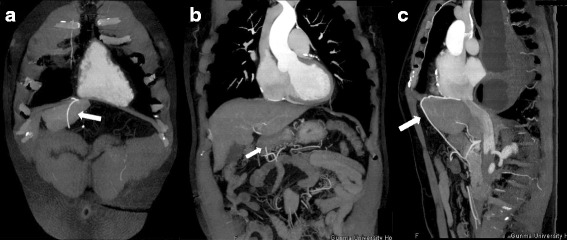
Three-dimensional CT findings. It is running through the front of the duodenal bulb and ventral of the hepatic lateral segment and going toward the back side beyond the diaphragm and reach to the RCA. **a**, **b** Coronal view. **c** Sagittal view. *White arrow* indicates the right gastroepiploic artery

**Fig. 6:**
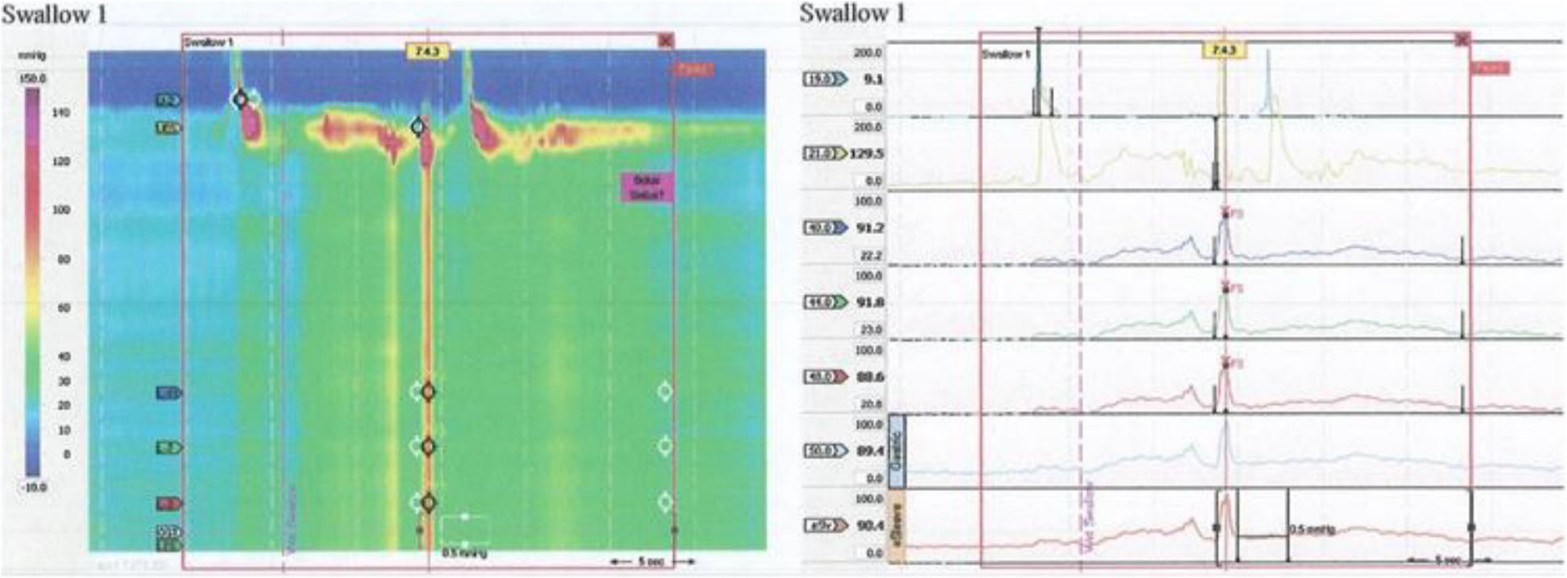
Esophageal manometry findings. The lower esophageal sphincter failed to relax, and the first peristaltic wave was absent

The patient was a 62-year-old man. Twelve years previously, he had undergone CABG for angina pectoris and a bypass between the right coronary artery and the RGEA had been constructed. Six years previously, he had been referred because of difficulty swallowing. He was diagnosed as having achalasia and treated with nicardipine hydrochloride and balloon dilatation in the Gastrointestinal Medicine Department of our hospital. He was referred to our surgical department again because he remained symptomatic, and we were consulted about surgical treatment for achalasia. Gastrointestinal endoscopy showed dilatation of the whole esophagus and food residues within it (Fig. 1). A barium esophagogram showed dilatation and meandering of the esophagus and delayed flow of barium into the stomach (Fig. 2). Enhanced computed tomography (CT) showed also dilatation of the esophagus and meandering of the lower esophagus (Fig. 3). Three-dimensional CT angiography showed the right gastroepiploic artery running to the right coronary artery (Figs. 4 and 5). Esophageal manometry showed failure of the lower esophageal sphincter to relax and absence of the first peristaltic wave (Fig. 6). Based on these findings, a decision was made to perform surgery.

We discussed the surgical procedure with cardiac surgeons preoperatively and ordered them to back up, considering the possibility of re-anastomosis at the time of the damage of the graft and the onset of cardiovascular complications. Additionally, we prepared papaverine hydrochloride against the graft spasm.

We also discussed the operative procedure in detail. It was expected that there is adhesion of intraperitoneal tissue because of the previous surgery. We could not deny presence of adhesion of the RGEA bypass graft and abdominal wall through the preoperative examination. Therefore, we decided to perform laparotomy in view of a risk of the graft damage at the time of the port insertion. Additionally, because it took time to cope with damage of the graft in laparoscopic surgery, we selected laparotomy after discussion with cardiac surgeons.

An oblique incision was made below the left costal arch in order to avoid the adhesion because of the previous surgery. The extent of adhesion was mild. Searching intraperitoneal at the time of laparotomy, we identified the RGEA graft wrapped by soft tissues due to palpation. The RGEA graft ran along the left edge of the round ligament of the liver and reached to the heart via the ventral and lateral side of the liver. We performed surgical procedure so as not to damage this graft while checking the graft beats by palpation appropriately. Especially when we operated the cardiac region, we performed carefully because we needed to retract the liver to secure the visual field. The second assistant dedicated to observation around the graft. After dissecting around the hiatus esophagus, the abdominal esophagus was pulled up by using a vessel tape and as much dissection as possible was performed around the cranial esophagus. The dilated esophagus was then pulled caudally and straightened under observation by gastrointestinal endoscopy (GIS). The vagal nerves, including the hepatic branch, were preserved. Myotomy was performed for 6 cm on the esophageal side and 2 cm on the gastric side (Heller myotomy) and dilation of the narrow segment observed via GIS. Having recognized that the fornix was mobile, a Dor fundoplication was performed. Additionally, two shoulder stitches were placed in the crus of the diaphragm. Finally, straightening and dilation of the esophagus was confirmed by GIS. The RGEA was not damaged during the procedure, and no fluctuation in circulatory dynamics or electrocardiographic changes occurred. On postoperative day 3, he started drinking water and on day 5 started feeding. His postoperative course was good, and he was discharged on day 12. Two months later, a barium esophagogram showed that the failure of the esophagogastric junction to relax had improved and barium was flowing smoothly into the stomach after deglutition. Postoperative esophageal manometry showed the stillness pressure of the esophagogastric junction had decreased, confirming the therapeutic effect of the operative procedure. At present, his symptoms are adequately controlled.

## Discussion

When performing upper abdominal surgery after CABG using RGEA, it is necessary to assess whether the graft is contributing to the coronary artery blood flow. If the graft is obstructed or coronary angiography shows a “to and fro” phenomenon despite the graft being patent, the graft is not contributing to the coronary artery blood flow and its inherent blood flow is being maintained. In such cases, standard lymphadenectomy with resection of the RGEA can be performed, for example, during gastrectomy for gastric cancer.

Conversely, if the graft is contributing to the coronary artery blood flow, injury or compression, including indirect injury or compression, may cause myocardial ischemia, coronary failure, and fatal arrhythmias. It has been reported that stretching of the abdominal wall can depress the ST segment in an electrocardiogram [2]. Additionally, mechanical and low-temperature stimulations can induce spasm in arterial grafts [2]. To avoid graft spasms, we sprinkle papaverine hydrochloride around the RGEA when performing gastrectomy after CABG.

To avoid graft injuries, it is necessary to assess the route of the graft by enhanced CT or abdominal angiography preoperatively: it can be difficult to identify the graft intraoperatively because of adhesions after CABG. In such cases, indocyanine green fluorescent angiography may help with identification of the graft.

It is also important to recognize how the graft for CABG has been procured. Two major techniques are employed, namely pedicle grafts that include surrounding soft tissues and skeletonized grafts that lack them. Pedicle grafts can be difficult to distinguish from the falciform ligament of the liver and mesentery, which may result in injury to a pedicle graft.

During both achalasia surgery and other abdominal surgeries, it is necessary to be careful not to injury the graft with a wound retractor used during laparotomy. There are two standard routes for RGEA grafts, namely an ante-gastric and a retro-gastric route; the latter is preferred by most cardiac surgeons [5]. Therefore, the part of the graft that is most vulnerable to injury during achalasia surgery is that in the cardiac region.

The other possible countermeasure is to first perform an angioplasty on the coronary artery. In one report of a patient having advanced gastric cancer after CABG using the RGEA, rerouting revascularization of the RGEA graft was performed via thoracoscopic surgery prior to gastric surgery [6]. In any case, it is necessary to discuss the surgical procedure with cardiac surgeons preoperatively and prepare for possible contingencies.

To our knowledge, this is the first reported case of surgery for achalasia performed after CABG using the RGEA. Surgical procedures for achalasia comprise Heller–Dor surgery (laparotomy or laparoscopic) and per-oral endoscopic myotomy [7]. In our case, we considered that per-oral endoscopic myotomy would be a safer approach. However, because this patient’s esophagus was dilated and meandering in a sigmoid pattern, we needed to linearize it prior to performing a myotomy; we therefore chose the Heller–Dor procedure. Additionally, we considered laparotomy safer than laparoscopic surgery from the perspective of preserving the RGEA. Laparoscopic surgery has advantages in that if the ports are inserted in adequate sites, we can perform procedures while keeping distance from RGEA graft and there is little stress except the port site. Conversely, it has disadvantages in that visual field is limited, and there is possibility of damaging the graft by taking forceps in and out, and it takes time to cope with damage of the graft. On the other hand, laparotomy has advantages in that because it provides wide visual field, we can perform the procedure while including the graft in the visual field and checking beats palpation.

For these reasons, we believed that Heller–Dor via laparotomy was the procedure of choice for this patient.

## Conclusions

We here present a case of surgery for achalasia after CABG with RGEA. When performing upper abdominal surgeries in such cases, it is necessary to investigate the patient carefully preoperatively and plan the intraoperative procedure so as to minimize risk of injury to the graft and consequent cardiovascular complications.

## Abbreviations

CABG: Coronary artery bypass graft
CT: Computed tomography
GIS: Gastrointestinal endoscopy
RGEA: Right gastroepiploic artery
